# Interactions of Environmental Factors and *APOA1-APOC3-APOA4-APOA5* Gene Cluster Gene Polymorphisms with Metabolic Syndrome

**DOI:** 10.1371/journal.pone.0147946

**Published:** 2016-01-29

**Authors:** Yanhua Wu, Yaqin Yu, Tiancheng Zhao, Shibin Wang, Yingli Fu, Yue Qi, Guang Yang, Wenwang Yao, Yingying Su, Yue Ma, Jieping Shi, Jing Jiang, Changgui Kou

**Affiliations:** 1 Department of Epidemiology and Biostatistics, School of Public Health, Jilin University, 1163 Xinmin Street, Changchun, 130021, Jilin province, China; 2 Division of Clinical Epidemiology, First Hospital of Jilin University, Changchun, Jilin, 130021, China; 3 Department of Endoscopy Center, China-Japan Union Hospital of Jilin University, Changchun, Jilin, 130021, China; Yale University, UNITED STATES

## Abstract

**Objective:**

The present study investigated the prevalence and risk factors for Metabolic syndrome. We evaluated the association between single nucleotide polymorphisms (SNPs) in the apolipoprotein *APOA1/C3/A4/A5* gene cluster and the MetS risk and analyzed the interactions of environmental factors and *APOA1/C3/A4/A5* gene cluster polymorphisms with MetS.

**Methods:**

A study on the prevalence and risk factors for MetS was conducted using data from a large cross-sectional survey representative of the population of Jilin Province situated in northeastern China. A total of 16,831 participations were randomly chosen by multistage stratified cluster sampling of residents aged from 18 to 79 years in all nine administrative areas of the province. Environmental factors associated with MetS were examined using univariate and multivariate logistic regression analyses based on the weighted sample data. A sub-sample of 1813 survey subjects who met the criteria for MetS patients and 2037 controls from this case-control study were used to evaluate the association between SNPs and MetS risk. Genomic DNA was extracted from peripheral blood lymphocytes, and SNP genotyping was determined by MALDI-TOF-MS. The associations between SNPs and MetS were examined using a case-control study design. The interactions of environmental factors and *APOA1/C3/A4/A5 g*ene cluster polymorphisms with MetS were assessed using multivariate logistic regression analysis.

**Results:**

The overall adjusted prevalence of MetS was 32.86% in Jilin province. The prevalence of MetS in men was 36.64%, which was significantly higher than the prevalence in women (29.66%). MetS was more common in urban areas (33.86%) than in rural areas (31.80%). The prevalence of MetS significantly increased with age (OR = 8.621, 95%CI = 6.594–11.272). Mental labor (OR = 1.098, 95%CI = 1.008–1.195), current smoking (OR = 1.259, 95%CI = 1.108–1.429), excess salt intake (OR = 1.252, 95%CI = 1.149–1.363), and a fruit and dairy intake less than 2 servings a week were positively associated with MetS (P<0.05). A family history of diabetes (OR = 1.630, 95%CI = 1.484–1.791), cardiovascular disease or cerebral diseases (OR = 1.297, 95%CI = 1.211–1.389) was associated with MetS. *APOA1* rs670, *APOA5* rs662799 and rs651821 revealed significant differences in genotype distributions between the MetS patients and control subjects. The minor alleles of *APOA1* rs670, *APOA5* rs662799 and rs651821, and *APOA5* rs2075291 were associated with MetS (*P<0*.*0016*). *APOA1* rs5072 and *APOC3* rs5128, *APOA5* rs651821 and rs662799 were in strong linkage disequilibrium to each other with r^2^ greater than 0.8. Five haplotypes were associated with an increased risk of MetS (OR = 1.23, 1.58, 1.80, 1.90, and 1.98). When we investigated the interactions of environmental factors and *APOA1/C3/A4/A5* gene cluster gene polymorphisms, we found that *APOA5* rs662799 had interactions with tobacco use and alcohol consumption (P_GE_<0.05).

**Conclusions:**

There was a high prevalence of MetS in the northeast of China. Male gender, increasing age, mental labor, family history of diabetes, cardiovascular disease or cerebral diseases, current smoking, excess salt intake, fruit and dairy intake less than 2 servings a week, and drinking were associated with MetS. The *APOA1/C3/A4/A5* gene cluster was associated with MetS in the Han Chinese. *APOA5* rs662799 had interactions with the environmental factors associated with MetS.

## Introduction

Metabolic syndrome (MetS) is a cluster of metabolic abnormalities that includes central obesity, high levels of triglycerides and glucose, low levels of high density lipoprotein, and elevated blood pressure [1]. MetS increases the risk of cardiovascular disease (CVD) and coronary artery disease (CAD) and serves as a risk factor for stroke [2–6].

The prevalence of MetS in the Chinese population was recently reported [7], especially in the south areas of China such as Jiangsu [8] and Zhejiang provinces [9] and in economic booming areas such as Beijing [10]. Jilin province is an underdeveloped area located in northeastern China that covers 780,000 square kilometers. For our study, we collected more than 16,000 samples from nine areas of Jilin province and investigated the prevalence and risk factors for MetS in Jilin province.

Several single nucleotide polymorphisms (SNPs) in different candidate genes (i.e., adipokine, lipoprotein, inflammation, adipose distribution, and glucose or energy metabolism candidate genes) have been associated with MetS [11]. Among these candidate genes, the Apolipoprotein *APOA1/C3/A4/A5* gene cluster on chromosome 11q23 is a lipoprotein candidate gene cluster that plays roles in plasma triglyceride metabolism and lipoprotein lipase activity [12–14].

Rs670 at *APOA1* is associated with higher HDL-C levels and higher diastolic or systolic blood pressure levels [15,16]. *APOC3* rs2854117 is associated with fatty liver and postprandial plasma TG [17,18]. *APOA4* has been reported to participate in lipoprotein lipase and lecithin cholesterol acyltransferase activity [19,20]. Rs5104 is not only a missense SNP but also a tag SNP at *APOA4*. *APOA5* rs662799 is correlated with dyslipidemia and postprandial lipoprotein metabolism [21–24]. According to several studies, *APOA5* rs662799 is associated with increased triglycerides, decreased HDL levels and MetS prevalence among Japanese individuals; however, no association between this SNP and MetS among Germans and Australians has been found [25,26].

In our study, we chose tag and well-studied SNPs located in the *APOA1/C3/A4/A5* gene cluster. Then, we tested the association of these SNPs with MetS in the Han Chinese population using a case-control study design and evaluated the interactions of environmental factors and *APOA1-APOC3-APOA4-APOA5* gene cluster polymorphisms with MetS.

## Materials and Methods

### Study population

This prevalence and risk factors study was conducted through a cross-sectional and representative survey of Jilin province, China. The study was designed to evaluate the prevalence and risk factors associated with chronic diseases [27]. The participants were randomly chosen from a multistage stratified cluster sampling of residents aged from 18 to 79 years in nine areas of Jilin province (both urban and rural areas) in 2012. In the first stage, the province was stratified into 9 regions, which cover all of Jilin Province. In the second stage, clusters of 4 districts or counties were randomly selected from each of the 9 regions using probability proportional to size (PPS) sampling. In the third stage, each selected district or county was divided into urban and rural areas as defined by the National Bureau of Statistics of China. Subsequently 4 or 5 communities were sampled from both rural and urban strata using PPS. Finally, 1 adult resident was randomly selected from each household of the selected communities. The loss of sampling was replaced only once. The response rate was 84.9%. A total of 16,831 participants (including 7,782 men and 9,109 women with an average age of 48 ± 13 years) who provided complete data for the variables of metabolic syndrome components were selected for this cross-sectional survey.

For the association and interaction study, 1,813 case subjects were randomly chosen using the SPSS by a method of simple random sampling from the patients with MetS in the survey above. Control subjects that were not affected with MetS were chosen from the same population that was surveyed for MetS at the same time. Both cases and controls were from the Han population in Northern China.

All subjects in this study signed a written informed consent form. Our study was approved by the ethics committee of the School of Public Health, Jilin University.

### Procedures, measurements, and quality control

All selected subjects were informed by the local health service centers using reservation cards prior to the beginning of the survey. Data were collected through face-to-face questionnaire interviews as well as anthropometry and laboratory examinations.

All interviews used the same structured questionnaire and were administered by well-trained investigators. The identification of the participants was checked prior to the interview, and the completeness of the questionnaire was checked after the interview. All participants provided informed consent. Demographic data, lifestyles, eating habits, family history, and chronic diseases were obtained by self-report. The educational levels were divided into pre-middle school, middle school, high school, and post-high school degree. The participants were employed in either manual labor in the agriculture, forestry, manufactory, and service industries or mental labor such as office clerks, government officials, professional staff in medicine and education, and full-time students. The smoking status was categorized into current smoker, former smoker, and never smoked. Participants who reported smoking at least 100 cigarettes in their lifetime and smoked either every day or on some days at the time of the survey were defined as current smokers. Participants who reported smoking at least 100 cigarettes in their lifetime and did not smoke at all at the time of the survey were defined as former smokers. Participants who reported never having smoked 100 cigarettes were defined as never smoked.

The anthropometry examinations included height, weight, waistline and hip circumference, blood pressure and heart rate. Height and weight were measured in subjects standing straight and wearing light clothing without shoes. The waistline and hip circumferences were separately measured at the level of the individual’s umbilicus and the maximum protrusion of the gluteal muscles, respectively. The instruments used for the measurements were from the same manufacturer and the same batch. The systolic and diastolic blood pressures were recorded with an average of two measurements in a sitting position after a 10-minute rest.

All subjects were asked to provide 5 mL of blood for biochemical analysis. The laboratory examinations included total cholesterol (TC), triglycerides (TG), high-density lipoprotein (HDL), low-density lipoprotein (LDL), and fasting glucose. The blood samples were collected in the morning after an overnight fast (at least 8 hours), transported to the same laboratory under refrigeration, and then stored at −20°C. The lab finished the blood examinations within 12 hours after receiving the blood samples and provided daily quality control charts. All data were double entered and validated.

### Identification of the metabolic syndrome (MetS)

The prevalence of MetS was evaluated according to the International Diabetes Federation criteria (2009). The identification of MetS was based on three or more of the following components: a) Central obesity: waist circumference ≥ 85 cm in men or ≥ 80 cm in women for Chinese subjects; b) Raised triglycerides: TG ≥ 1.7 mmol/L or undergoing treatment for hypertriglyceridemia; c) Low high-density lipoprotein levels: HDL-C < 1.04 mmol/L in men and <1.30 mmol/L in women or undergoing treatment; d) Elevated blood pressure: systolic blood pressure ≥ 135 mmHg, diastolic blood pressure ≥ 85 mmHg, or the use of antihypertensive drug therapy; and e) Raised glucose: fasting glucose ≥ 5.6 mmol/L or the use of antidiabetic drugs.

### SNP selection

Tag SNPs were selected using the Haploview program (http://hapmap.ncbi.nlm.nih.gov/). Three tag SNPs (*APOA1* rs5072, *APOC3* rs5128, and *APOA5* rs2075291) were obtained. Other well-studied or functional SNPs were selected based on previous studies that documented associations between SNPs in the Apolipoprotein *APOA1/C3/A4/A5* gene cluster and MetS or its components, including *APOA1* rs670, *APOC3* rs2854117, *APOA4* rs5104, *APOA5* rs662799, and *APOA5* rs651821. The minor allele frequency (MAF) of the above eight SNPs was greater than 0.05 in the Chinese Han population.

### DNA extraction and genotyping rate

Peripheral blood samples were collected in the morning using non-anticoagulant plexiglass tubes and stored at −20°C. Genomic DNA was extracted from peripheral blood lymphocytes using a commercial DNA extraction kit (Hangzhou, China). SNP genotyping was determined by MALDI-TOF-MS (Sequenom, San, Diego, CA, USA) using the MassARRAY system. Completed genotyping reactions were dispensed onto a 384-well spectro CHIP using a MassARRAY Nanodispenser (Sequenom)[28].

The detection rates for the SNPs *APOA1* rs670 and rs5072, *APOC3* rs5128 and rs2854117, *APOA4* rs5104, *APOA5* rs662799 and rs651821, and *APOA5* rs2075291 were 91.4%, 99.7%, 99.6%, 98.3%, 95.8%, 99.1%, 99.6%, and 95.9%, respectively.

### Statistical analysis

The statistical analyses were conducted using the SPSS system (version 19.0). The prevalence of MetS was adjusted by a complex weighted computation with the gender, age and location distribution according to the sixth Chinese National Population Census, 2010 [29].

Rao-Scott χ2 test was used to compare the prevalence of MetS between different genders and locations. MetS risk factors were examined using uni- and multivariate logistic regression analyses based on the complex weighted computation. The minor allele frequency of the SNPs was compared between subjects with and without MetS using the χ^2^ test. For each SNP, the Hardy-Weinberg disequilibrium (HWD) test was conducted in the control group. Associations between the SNPs and MetS were examined by multiple logistic regression analyses after adjusting for age and gender. There were four different models of inheritance in our study. G and A represent a pair of alleles. If A is a minor allele, the dominant model indicates GG vs AG+AA, the recessive model indicates AA vs AG+GG, the codominant model indicates GG vs GA vs AA, and the overdominant or super dominant model indicates GG + AA vs GA. The best model of inheritance for the SNPs was selected based on the Akaike information criterion (AIC), the Haplotype estimation and association were using the SNPStats program (http://bioinfo.iconcologia.net/SNPStats) [30], and the model with the minimum AIC value was the best model. A Bonferroni correction was used to account for multiple statistical tests for each SNP association. A *P*<0.05/32 = 0.0016 was selected as the appropriate significance level.

The interactions between environmental factors and *APOA1-APOC3-APOA4-APOA5* gene cluster gene polymorphism with MetS were assessed using multivariate logistic regression analysis. For positive interaction findings the online software Quanto was used to estimate statistical power (http://hydra.usc.edu/GxE/).

## Results

### The Prevalence of MetS

A total of 16,831 participants were included in the study. The participants ranged in age from 18–79 years with a mean age of 48 years (SD = 13). A total of 15,492 (92.0%) of the participants were Han people. The participants came from rural (48.6%) and urban (51.4%) areas across Jilin Province.

Table 1 showed that the overall prevalence of MetS was 32.86% (SE = 0.44%). The prevalence of MetS in men was 36.64% (SE = 2.30%), which was significantly higher than the prevalence in women of 29.66% (SE = 0.59%). However, the prevalence of Mets was higher in women than in men in the age groups 55–64 and 65–79 years old. MetS was significantly more common in the urban areas than in the rural areas (P < 0.05). Regardless of whether the comparisons were made between the male and female groups or the urban and rural groups, the prevalence of MetS significantly increased with age.

**Table 1:** Prevalence of MetS using IDF definition with age in different gender and location among Jilin province, China.

Age group	Overall	Sex		Location	
		Male	Female	Urban	Rural
18–24	8.59±1.21	11.06±1.61	6.61±1.78*	7.80±1.24	9.34±2.06 ^†^
25–34	21.18±1.00	31.42±1.60	11.33±1.12*	21.55±1.31	20.71±1.55^†^
35–44	30.41±0.77	41.16±1.25	21.25±0.90*	30.90±1.09	29.89±1.10^†^
45–54	42.53±0.74	46.91±1.13	38.85±0.98*	43.74±1.08	41.24±1.02^†^
55–64	50.21±0.87	44.25±1.34	54.97±1.14*	53.02±1.35	47.52±1.10^†^
65–79	51.88±1.53	39.08±2.30	61.84±1.76*	55.42±2.44	47.73±1.78^†^
Total	32.86±0.44	36.64±2.30	29.66±0.59*	33.86±0.60	31.80±0.65^†^

Data were shown as frequency (% subjects) ±SE

Prevalence were adjusted by complex weighted computation with the sex, age and location distribution according to the tabulation on the 2010 population census of Jilin province of China's Sixth Population Census(16).

*P<0.05, Rao-Scott χ^2^ test for the prevalence of MetS between male and female.

^†^P<0.05, Rao-Scott χ^2^ test for the prevalence of MetS between urban and rural.

### Factors associated with MetS

The uni- and multivariate logistic regression analysis results are shown in Table 2. Women had a higher prevalence of MetS than men. An older age, low educational level, retirement or unemployment, former smoker, higher dietary meat intake, excess salt intake, and fruit and dairy intake less than two servings a week were positively associated with MetS. People with a family history of diabetes, cardiovascular or cerebral diseases had a higher risk of MetS (P < 0.05).

**Table 2:** Univariate and multivariate logistic regression results for the factors associated with MetS as defined by IDF among adults in Jilin Province, China.

Variables		Univariate			Multivariate		
		OR	95CI%	P value	OR	95CI%	P value
Gender	Male	1			1		
	Female	0.729	0.674–0.788	<0.001	0.705	0.622–0.799	<0.001
Age	18–24	1			1		
	25–34	2.860	2.066–3.959	<0.001	3.353	2.266–4.961	<0.001
	35–44	4.652	3.407–6.352	<0.001	5.212	3.549–7.655	<0.001
	45–54	7.878	5.784–10.730	<0.001	8.659	5.893–12.723	<0.001
	55–64	10.738	7.870–14.652	<0.001	11.673	7.825–17.413	<0.001
	65—	11.478	8.285–15.902	<0.001	13.995	9.157–21.391	<0.001
Location	Urban	1			—		
	Rural	0.911	0.842–0.985	0.020			
Education	Less than middle school	1			1		
	Lower-middle school degree	0.753	0.681–0.834	<0.001	0.921	0.805–1.053	0.228
	Higher-middle school degree	0.894	0.807–0.990	0.031	0.887	0.736–1.070	0.210
	Post Higher-middle school	0.609	0.540–0.688	<0.001	0.865	0.751–0.996	0.043
Employment	Manual labor	1			1		
	Brain labor	0.952	0.860–1.055	0.350	1.091	0.950–1.252	0.216
	Retired/unemployment	2.080	1.869–2.316	<0.001	1.508	1.312–1.733	<0.001
Alcohol consumption	No	1			—		
	Yes	1.235	1.138–1.340	<0.001			
Smoking	Never	1			1		
	Current	1.337	1.227–1.458	<0.001	1.039	0.914–1.180	0.559
	Former	2.123	1.857–2.427	<0.001	1.323	1.109–1.578	0.002
Diets	Balance	1			1		
	Meat more	1.450	1.259–1.669	<0.001	1.422	1.202–1.684	<0.001
	Vegetable more	1.209	1.109–1.317	<0.001	0.980	0.880–1.092	0.718
Salt intake	Less intake	1			1		
	Normal intake	0.823	0.744–0.911	<0.001	1.002	0.890–1.128	0.969
	Excess intake	1.129	1.027–1.241	0.001	1.229	1.102–1.370	<0.001
Nutrition intake ^a^	Fruit:< 2 times/w	1.653	1.506–1.814	<0.001	1.165	1.036–1.410	0.011
	Aquatic product:< 2 times/w	0.988	0.914–1.069	0.077	0.924	0.840–1.017	0.055
	Red meet:< 2 times/w	1.305	1.191–1.429	<0.001	—		
	Egg: <2 times/w	1.016	0.891–1.158	0.811	—		
	Dairy: < 2 times/w	1.695	1.560–1.842	<0.001	1.308	1.174–1.456	<0.001
Family history ^b^	Cardiovascular or cerebral diseases	1.272	1.176–1.375	<0.001	1.334	1.206–1.476	<0.001
	Diabetes	1.427	1.277–1.375	<0.001	1.553	1.377–1.752	<0.001
	Respiratory system diseases	1.118	1.012–1.236	0.029	—		
	Hyperlipidemia	1.186	0.978–1.437	0.082	—		

OR: odds ratio; CI: confidence interval;

^a^ The references for each type of nutrition intake was frequency ≥2 times/w.

^b^ The references for each type of family history was none.

### Allele frequency comparisons

The distributions of eight SNPs (*APOA1* rs670 and rs5072, *APOC3* rs5128 and rs2854117, *APOA4* rs5104, *APOA5* rs662799 and rs651821, and *APOA5* rs2075291) were in Hardy-Weinberg equilibrium (HWE) in the non-MetS group. The comparisons of genotype distributions and allele frequencies of the polymorphisms in the *APOA1/C3/A4/A5* gene cluster between subjects with and without MetS are shown in Table 3. Three SNPs (*APOA1* rs670, *APOA5* rs662799 and rs651821) revealed significant differences in genotype distributions between subjects with and without MetS. The frequencies of the *APOA5* rs651821 C allele, *APOA5* rs662799 G allele, and *APOA5* rs2075291 T allele were significantly higher in the non-MetS group compared to the MetS group (P < 0.001). However, allele frequency differences between the MetS and the non-MetS groups were not observed for *APOA1*rs5072, *APOC3* rs5128 and rs2854117, and *APOA4* rs5104.

**Table 3:** Comparison of genotype distributions and allele frequencies of polymorphisms in the *APOA1/C3/A4/A5* gene cluster between subjects with and without metabolic syndrome.

Gene	Rs number	Genotype /allele	Metabolic Syndrome(%)	Non-Metabolic Syndrome(%)	P value
*APOA1*	rs670	GG	934(55.9)	1106(62.5)	<0.001
		AA	143(8.6)	76(4.3)	
		GA	593(35.5)	589(33.3)	
		G allele	2461(73.7)	2801(79.1)	<0.001
		A allele	879(26.3)	741(20.9)	
	rs5072	TT	170(9.4)	203(10.0)	0.038
		CC	801(44.5)	978(48.0)	
		TC	831(46.1)	856(42.0)	
		T allele	1171(32.5)	1262(31.0)	0.177
		C allele	2442(67.5)	2812(69.0)	
*APOC3*	rs5128	GG	815(45.3)	1004(49.3)	0.008
		CC	151(8.4)	191(9.4)	
		GC	833(46.3)	841(41.3)	
		G allele	2463(68.5)	2849(70.0)	0.152
		C allele	1135(31.5)	1223(30.0)	
	rs2854117	GG	560(31.5)	676(33.6)	0.348
		AA	330(18.6)	351(17.5)	
		GA	886(49.9)	983(48.9)	
		G allele	2006(56.5)	2335(58.1)	0.158
		A allele	1546(43.5)	1685(41.9)	
*APOA4*	rs5104	GG	169 (9.8)	198(10.1)	0.067
		AA	791(45.7)	962(49.1)	
		GA	771(44.5)	799(40.8)	
		G allele	2353(68.0)	2723(69.5)	0.156
		A allele	1109(32.0)	1195(30.5)	
*APOA5*	rs662799	CC	194(10.9)	118(5.8)	<0.001
		TT	827(46.3)	1145(56.4)	
		TC	767(42.9)	766(37.8)	
		C allele	1155(32.3)	1002(24.7)	<0.001
		T allele	2421(67.7)	3056(75.3)	
	rs651821	TT	833(46.3)	1145(56.2)	<0.001
		CC	194(10.8)	116(5.7)	
		TC	772(42.9)	775(38.1)	
		T allele	2438(67.8)	3065(75.2)	<0.001
		C allele	1160(32.2)	1007(24.8)	
	rs2075291	GG	1507(86.8)	1768(90.3)	0.003
		TT	8(0.5)	4(0.2)	
		GT	220(12.7)	185(9.5)	
		G allele	3234(93.2)	3721(95.1)	<0.001
		T allele	236(6.8)	193(4.9)	

### Genotype distribution comparison

Table 4 shows the multiple logistic regression analysis results of the best selected model after adjusting for the confounding factors age and gender. Inheritance modeling suggested an overdominant model for *APOA1* rs5072, *APOC3* rs5128, and *APOA4* rs5104, a dominant model for *APOC3* rs2854117 and *APOA5* rs2075291, and a codominant model for *APOA1* rs670, *APOA5* rs651821, and *APOA5* rs662799. The genotypes of *APOA1* rs670, *APOC3* rs5128, *APOA5* rs662799 and rs651821, and *APOA5* rs2075291 were associated with MetS (*P <0*.*0016*).

**Table 4:** Genotype distribution comparisons and odds ratio (OR) estimates of eight SNPs in the *APOA1/C3/A4/A5* gene cluster.

Gene	Rs number	Genotype	Inheritance model	Non-Metabolic Syndrome(%)	Metabolic Syndrome(%)	Adjusted OR (95% CI)^a^	*P* value
*APOA1*	rs670	GG	Codominant	934 (55.9%)	1106 (62.5%)	1.00	<0.001
		GA		593 (35.5%)	589(33.3%)	1.19 (1.03–1.38)	
		AA		143 (8.6%)	76 (4.3%)	2.23 (1.67–2.98)	
	rs5072	CC/TT	Over dominant	971 (53.9%)	1181 (58.0%)	1.00	0.007
		TC		831 (46.1%)	856 (42.0%)	1.18 (1.04–1.34)	
*APOC3*	rs5128	GG/CC	Over dominant	966 (53.7%)	1195 (58.7%)	1.00	0.001
		GC		833 (46.3%)	841 (41.3%)	1.23 (1.08–1.39)	
	rs2854117	GG	Dominant	560 (31.5%)	676 (33.6%)	1.00	0.170
		AG/AA		1216 (68.5%)	1334 (66.4%)	1.10 (0.96–1.26)	
*APOA4*	rs5104	AA/GG	Over dominant	960 (55.5%)	1160(59.2%)	1.00	0.021
		GA		771 (44.5%)	799 (40.8%)	1.17 (1.02–1.33)	
*APOA5*	rs662799	TT	Codominant	827 (46.2%)	1145 (56.4%)	1.00	<0.001
		TC		767 (42.9%)	766 (37.8%)	1.39 (1.21–1.59)	
		CC		194 (10.8%)	118 (5.8%)	2.28 (1.78–2.91)	
	rs651821	TT	Codominant	833 (46.3%)	1145 (56.2%)	1.00	<0.001
		TC		772 (42.9%)	775 (38.1%)	1.37 (1.20–1.57)	
		CC		194 (10.8%)	116 (5.7%)	2.30 (1.80–2.95)	
	rs2075291	GG	Dominant	1507 (86.9%)	1768 (90.3%)	1.00	<0.001
		GT/TT		228(13.1%)	189 (9.7%)	1.42 (1.15–1.74)	

^a^OR was adjusted for age and gender

### Linkage disequilibrium (LD) and haplotype analyses

**Fig 1:**
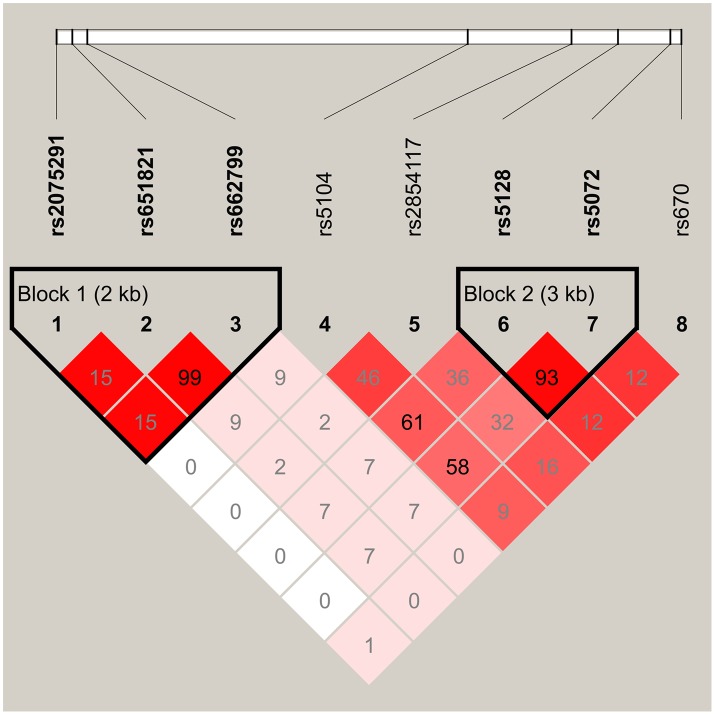
Linkage disequilibrium of the SNPs in the APOA1/C3/A4/A5 gene cluster. The color scale ranges from red to white (color intensity decreases with decreasing r^2^ value). This plot was generated by Haploview (version 4.1).

The pattern of pairwise linkage disequilibrium (LD) between the SNPs among *APOA1/C3/A4/A5* gene cluster was shown in Fig 1. *APOA1* rs5072 and *APOC3* rs5128, *APOA5* rs651821 and rs662799 were in strong linkage disequilibrium to each other with r^2^ greater than 0.8.

The haplotype analysis identified five risk haplotypes that were significantly associated with an increased risk of MetS (OR = 1.23, 1.58, 1.80, 1.90, and 1.98) (Table 5).

**Table 5:** Associations between *APOA1/C3/A4/A5* Gene cluster haplotypes and risk of metabolic syndrome.

Haplotype	SNP^a^								Frequency			Adjusted OR (95% CI)^b^	P value
	1	2	3	4	5	6	7	8	Total	Controls	Cases		
1	G	C	G	G	A	T	T	G	0.2652	0.2863	0.2423	1.00	---
2	A	C	G	G	A	T	T	G	0.1430	0.1406	0.1466	1.23 (1.04–1.46)	0.017
3	G	T	C	A	G	T	T	G	0.1235	0.1334	0.1132	0.99 (0.83–1.17)	0.900
4	G	T	C	A	G	C	C	G	0.1085	0.0949	0.1244	1.58 (1.33–1.89)	<0.001
5	G	C	G	A	A	T	T	G	0.1063	0.1072	0.1047	1.19 (0.99–1.42)	0.058
6	A	C	G	G	A	C	C	G	0.0511	0.0406	0.0612	1.80 (1.39–2.33)	<0.001
7	G	C	G	G	A	C	C	G	0.0225	0.0256	0.0215	1.04 (0.68–1.60)	0.850
8	G	C	G	A	G	T	T	G	0.0204	0.0229	0.0173	0.85 (0.56–1.28)	0.430
9	A	C	G	G	A	C	C	T	0.0193	0.0151	0.0234	1.90 (1.26–2.86)	0.002
10	G	T	C	G	A	T	T	G	0.0190	0.0203	0.0157	1.00 (0.66–1.52)	0.990
11	G	C	G	G	A	C	C	T	0.0126	0.0123	0.0116	1.29 (0.77–2.16)	0.340
12	G	T	C	A	G	C	C	T	0.0103	0.0077	0.0132	1.98 (1.10–3.58)	0.023
Rare^c^	-	-	-	-	-	-	-	-	0.0983	0.0931	0.1049	1.33 (1.08–1.63)	0.007

^a^ SNP are as follows: 1, *APOA1*rs670; 2, *APOA1*rs5072; 3, *APOC3*rs5128; 4, *APOC3*rs2854117; 5,*APOA4*rs5104; 6, *APOA5*rs651821; 7, *APOA5*rs662799; 8,*APOA5*rs2075291.

^b^ Odds ratio (OR) was adjusted for age and sex.

^c^ Rare: haplotypes with frequencies<0.01.

### Interaction analysis

We used portions of the positive variables from the risk factors study as the environmental variables and the haplotype from the association study as the gene variables. Only *APOA5* rs662799 had interactions with tobacco use and alcohol consumption (P_EG_<0.05). *APOA5* rs662799 had a positive interaction with tobacco use or alcohol consumption (OR_EG_ = 3.54) (Table 6).

**Table 6:** Logistic regression of interaction between *APOA5* rs662799 polymorphism and environmental risk factors^a^.

Interaction terms	Factor	*χ*^2^	P	OR	95% CI	
					Lower	Upper
Smoking	G	11.332	0.001	1.330	1.126	1.570
	E	2.858	0.091	0.854	0.711	1.026
	EG	5.271	0.022	1.357	1.046	1.761
	Constant	19.309	<0.001	0.773		
Alcohol	G	14.715	<0.001	1.355	1.160	1.583
	E	1.311	0.252	1.118	0.924	1.353
	EG	5.615	0.018	1.394	1.059	1.836
	Constant	40.874	<0.001	0.701		

^a^ Positive result only

## Discussion

Our study was a community-based cross-sectional study with a large representative sample in Jilin Province, northeast China. The prevalence of MetS using the IDF definition in Jilin Province was 32.9% (SE = 0.4%). This prevalence was lower than the prevalence reported in the U.S. (39.0%) [31] and European (35.0% for men and 34.1% for women from the DECODE Study Group) populations [32]. The prevalence of MetS in Jilin Province was the same as the prevalence in Koreans (32.5% for men and 31.8% for women over 30 years of age) [33] but was higher than in the prevalence in the Japanese population (13.9%) [34]. The prevalence of MetS in Jilin Province was also higher than the prevalence in southern China reported for Jiangsu Province (30.5%)[8], Guangdong Province (26.7%)[35], and Taiwan (14.3%) [36]. Compared with the results from another epidemiological survey of MetS in Songyuan (an area of Jilin province) in 2010 [37], our study also showed a trend of increased prevalence of MetS in Jilin province (from 22.4% in 2009 to 32.86% at present).

In most published studies, women had a similar or a much higher prevalence of MetS than men [31,32,34,35]. In the present study, the prevalence of MetS was higher in men than in women except in the age groups 55–64 and 65–79. This discrepancy may be due to a higher prevalence of obesity, hypertension, low HDL-cholesterol and high TG levels in men compared to women prior to the age of 55. After 55 years of age, menopausal or postmenopausal women produced less estrogen, leading to changes in metabolism. The univariate logistic regression analysis results suggested that the prevalence of MetS among individuals in urban areas was much higher than the prevalence in rural areas. One possible explanation for this finding was that the prevalence of obesity and low HDL-cholesterol were higher in the urban areas. Additionally, the results indicated that age was positively associated with the MetS risk, possibly because the prevalence of hypertension, diabetes and dyslipidemia increased with age.

The multivariate logistic regression analysis results suggested that poor education and retirement or unemployment were associated with MetS. The level of education can impact an individual’s cognitive status; thus, individuals employed in a mental labor field may have a higher level of education and an increased awareness of the disease.

In the present study, alcohol consumption did not show a significant effect on MetS based on the multivariate logistic regression. However, the univariate logistic regression analysis demonstrated an impact of alcohol consumption on the risk of MetS. One possible explanation for the inconsistent result is that alcohol consumption influences the risk of MetS in a gender-specific manner. Current smoking did not have a significant effect on MetS in the multivariate logistic regression analysis; however, former smoking did have a significant effect. One explanation for this results is that individuals with chronic diseases such as hypertension may have given up smoking.

The relationship between nutrition intake and MetS has been well established. Our results indicated that daily excess, high meat intake, and excess salt intake were associated with the risk of MetS by increasing risk components of MetS such as central obesity, dyslipidemia or hypertension. In our study, a dairy intake of less than 2 servings a week was a risk factor for MetS. This result concurred with findings from Mexican, European, and Middle Eastern populations in which individuals with a higher dairy consumptions had a lower prevalence of MetS [38,39][40][41]

Genes correct physiological processes. Their expression patterns may result in metabolic abnormalities that contribute to disease processes. Family history may reflect genetic susceptibility to the disease. As a result, a family history of specific diseases, such as cardiovascular disease, cerebral diseases, and diabetes, can have an effect on MetS by influencing MetS components.

Previous studies demonstrated that SNPs located in the *APOA1*/*C3*/*A4*/*A5* gene cluster were associated with metabolic syndrome (MetS), insulin resistance, and cardiovascular disease in several ethnic populations [42]. However, the contribution of these SNPs to MetS in the Han Chinese is unknown.

*APOA1* encodes apolipoprotein A-I, which is the major protein constituent of high density lipoprotein (HDL)[43]. Mounting evidence suggests that *APOA1* influences plasma lipoprotein levels [44]. In the present study, we found that the frequency of the *APOA1* rs670 A allele was higher in the Chinese Han population than in populations from western countries [45]. However, the frequency of the *APOA1* rs670 A allele did not significantly differ between the MetS group and non-MetS group. Several studies showed that the A allele of *APOA1* rs670 was associated with HDL-C levels and that the association was either gender- or age-dependent [46,47]. The present study showed that subjects carrying the GA and AA genotypes of *APOA1* rs670 had an increased risk of developing MetS compared to subjects with the GG genotype (*P* < 0.001). Rs5072 was a tag SNP in *APOA1* with a MAF of 0.363 in the Han Chinese. In another study, *APOA1* rs5072 was associated with MetS in South Asian immigrants in the USA [48]. For this SNP, our data suggested an overdominant model of inheritance. After a Bonferroni correction, rs5072 was not associated with MetS (adjusted OR = 1.19; 95% CI, 1.05–1.35; *P* = 0.007). The Bonferroni method is the most conservative correction for multiple comparisons of SNPs but comes at the cost of increasing the probability of producing false negatives and consequently reducing the statistical power.

*APOC3*, which is synthesized by the liver and intestine, is an important marker of TG-rich lipoproteins levels [49]. A previous study showed that the overexpression of the *APOC3* gene resulted in higher triglyceride levels [50]. Moreover, *APOC3* was transcriptionally down-regulated by insulin levels [51]. Several studies revealed that *APOC3* rs5128 was associated with increased plasma TG levels [23,52,53]. The frequency of the *APOC3* rs5128 G allele in our study subjects was higher than that of Indian and Japanese populations [54,55]. The distribution of the G or C allele of rs5128 was not significantly different between the MetS group and the non-MetS group. Inheritance modeling suggested an overdominant model for *APOC3* rs5128. Subjects with the rs5128 genotype GC had an increased risk of MetS compared to subjects carrying the GG or CC rs5128 genotypes. Although previous studies demonstrated that *APOC3* rs2854117 was associated with fatty liver and postprandial plasma TG levels [17,56], we did not observe significant differences in either the allele frequency or the genotype distribution of *APOC3* rs2854117 between the MetS group and the non-MetS group in the present study.

*APOA4* is a potent activator of lecithin-cholesterol acyltransferase *in vitro*. The *APOA4* gene (*APOA4*) contains three exons and two introns. Because the function of *APOA4* is unclear, few studies have examined the association of polymorphisms in *APOA4* with MetS. Rs5104 in *APOA4* was the only tag SNP with a MAF greater than 0.05 in the Han Chinese. In the present study, the allele frequencies of *APOA4* rs5104 were not significantly different between subjects with and without MetS, and the rs5104 genotype was not associated with MetS after a Bonferroni correction.

The *APOA5* gene is the newest identified member in the *APOA1/C3/A4/A5* gene cluster region by Pennacchio et al. and van der Vliet et al. [57,58]. *APOA5* variants have been associated with triglyceride, HDL-C, and total plasma cholesterol levels [59]. Among the eight SNPs, *APOA5* rs662799 was the most widely studied in recent years. Several studies demonstrated either a gender- or ethnic-specific association of this SNP with MetS [26,60]. One meta-analysis showed that individuals in European and Chinese populations with the C allele of *APOA5* rs662799 had a 33% and 40% increased risk of developing MetS, respectively [61]. In our study, the frequency of the C allele of *APOA5* rs662799 in MetS patients was significantly higher than the frequency in the control subjects. We found that carriers of the CT genotype of rs662799 had a 1.38-fold increased risk of developing MetS compared to subjects with genotype TT and that carriers of the CC genotype of this SNP had a 2.26-fold increased risk of developing MetS compared to subjects with genotype TT. We also found that the TC and TT genotypes of rs651821 and the GG or TT genotype of rs2075291 were associated with an increased risk of MetS. Moreover, *APOA5* rs651821 was found to be in strong linkage disequilibrium with *APOA5* rs662799. Yin et al. reported that the *APOA1/C3/A5* haplotypes were associated with serum lipid levels in a Chinese population [12]. The results from another study suggested that two haplotypes of the *APOA1/C3/A5* gene cluster were associated with an increased risk of hypertriglyceridemia in a Taiwanese population [13]. However, the associations between haplotypes of the *APOA1*/*C3*/*A4*/*A5* gene cluster and MetS have not been examined in the Chinese population. In our study, five distinct haplotypes (haplotypes 2, 4, 6, 9, and 12; Table 5) from the 12 haplotypes of the *APOA1*/*C3*/*A4*/*A5* gene cluster were significantly associated with the risk of MetS in the Han Chinese population. Our results suggested that haplotypes 6, 9, and 12 were stronger predictive factors for MetS. These three haplotypes all contained the same alleles of *APOA5* rs651821 and rs662799. It is possible that the SNPs in *APOA5* may have a larger effect on the susceptibility to MetS.

One interesting finding in our study was the interaction effect of *APOA5* rs662799 between tobacco use or alcohol consumption. *APOA5* rs662799 had a positive interaction with tobacco use or alcohol consumption whether in additive model or multiplitive model. The power of our case/control samples based on 1807/2043 sample size to detect G×E interactions was estimated more than 60.15%. In another study, researchers found that group with the *APOA5* rs662799 CT and CC genotypes showed higher levels of TG than the group of TT genotype in the ex-smokers and current smokers, but not in the non-smokers [62], and TG level was one of the components of MetS. Other studies found that drinking were positive associated with HDL-C and TG [63,64]. RX Yin [65] and colleagues found that *APOA5* rs662799 had interaction effect with alcohol consumption on serum lipid levels. These interactions may contribute to the interaction effect between *APOA5* rs662799 and alcohol consumption on MetS. According to our result, cutting out smoking and drinking could be recommended in the people with CT and CC genotypes of *APOA5* rs662799.

The innovations of this study include: a) the participants in the association study were recruited from the community-based population; therefore, the data can be applicable to the general population and b) the sample size was sufficiently large in our study to ensure that we obtained strong statistical power. Our study also had some limitations. The prevalence of MetS was determined using a cross-sectional survey. Therefore, although the results in our study may not accurately reflect the causal relationship between the risk factors and MetS, they provided information on the association between them. In this study, the information obtained concerning the subjects’ diets was not sufficient to discuss the relationship between the diet and MetS. We selected only tag SNPs with a MAF greater than 0.05 in the Han Chinese and SNPs reported in published articles. Therefore, we may have neglected some functional SNPs in the *APOA1*/*C3*/*A4*/*A5* gene cluster. We found that *APOA5* rs662799 had a positive interaction with tobacco use or alcohol consumption (OR_EG_ = 3.54). Therefore, interventions in smoking behavior and alcohol consumption should be designed for people who carry the genotypes AG+GG.

## Conclusions

In summary, there was a high prevalence of MetS in the northeast of China. The risk of MetS increased significantly with age. The male gender, lower education, retirement or unemployment, former smoker, higher dietary meat intake, excess salt intake, fruit or dairy intake less than 2 servings a week, and a family history of diabetes, cardiovascular or cerebral diseases were positively associated with MetS. The results presented here showed that the *APOA1*/*C3*/*A4*/*A5* gene cluster was associated with MetS. *APOA5* rs662799 had interactions with the environmental factors that contributed to MetS.
